# Adjacent tooth trauma in complicated mandibular third molar surgery: Risk degree classification and digital surgical simulation

**DOI:** 10.1038/srep39126

**Published:** 2016-12-15

**Authors:** Zhou-Xi Ye, Chi Yang, Jing Ge

**Affiliations:** 1 Shanghai Stomatological Hospital, Fudan University, Shanghai, People’s Republic of China; 2 Department of Oral and Maxillofacial Surgery, Ninth People’s Hospital, College of Stomatology, Shanghai Jiao Tong University School of Medicine, and Shanghai Key Laboratory of Stomatology, Shanghai, People’s Republic of China

## Abstract

Analysis of adjacent tooth resistance is essential in wisdom teeth extraction to prevent adjacent tooth trauma, however it lacks adequate attention nowadays. This study aims at suggesting special extraction methods based on adjacent tooth resistance analysis for prevention of adjacent tooth damage. In this study, 136 complicated mandibular third molars extracted using piezosurgery were reviewed and classified based on the adjacent teeth resistances shown in orthopantomogram (OPG) during their mesio-distal rotations: degree I refers to teeth with no adjacent teeth resistance; degree II refers to teeth with resistance released after mesial-half crown sectioning; degree III refers to teeth which still had resistance after mesial-half crown sectioning. With the use of surgical simulations using cone beam computerized tomography (CBCT) reconstruction, all teeth in degree I were designed to rotate mesio-distally; 86.36%(38/44) teeth in degree II were designed to rotate mesio-distally after mesio-half crown sectioning; 69.09%(36/55) teeth in degree III were designed to rotate bucco-lingually. All teeth were extracted successfully, and only one adjacent tooth was subluxated due to the incomplete bone removal. Our study suggested that in order to prevent adjacent teeth trauma, complete bone removal is of importance, and impacted teeth with higher adjacent teeth trauma risks should consider bucco-lingual rotations.

Risk of adjacent tooth trauma in mandibular third molar extraction refers to the possibility of adjacent tooth damage (looseness or dislocation)^1^,^2^ during mesio-distal rotation of the impacted tooth. The risk of this trauma is often proportional to the adjacent tooth resistance. However, according to our knowledge, this complication has not arisen adequate attention due to the misdiagnosis and failure to report.

The prevention measures for this complication include bone removal and tooth sectioning^3^. Compared with the chisel and drill, the piezosurgery is the best to be introduced in complicated wisdom tooth surgery with higher accuracy and safety^4^,^5^,^6^. Tooth sectioning could also help release adjacent tooth resistance^7^. However, the inappropriate force of tooth sectioning leads to inferior alveolar nerve (IAN) damage or mandible fracture. Therefore, tooth sectioning should be considered as a supplementary measure after enough bone removal in complicated wisdom tooth extraction. The main problems are: when extracting teeth in different risk degrees, how do the two measures “cooperate” to prevent adjacent tooth trauma ? Cone beam computerized tomography (CBCT) and its reconstruction are popular applied in oral and maxillofacial surgery nowadays^8^. Is it possible to introduce this technique to guide the extraction ? To answer those questions, we designed a risk degree classification and presented different extraction methods based on it.

## Material and Methods

### Patients

This is a retrospective study. This study followed the Declaration of Helsinki on medical protocol and ethics and the regional Ethical Review Board of Shanghai Ninth People’s Hospital approved the study. All patients were informed about surgical purpose, surgical protocol, recovery period, possible complications and signed a consent form.

From May 1^st^ 2012 to May 1^st^ 2015, 113 cases with 136 complicated mandibular third molars extracted using piezosurgery by the same surgeon (Chi Y) were reviewed. The surgeon was a chief physician with 30-year clinical experience. The inclusion criteria were the complicated mandibular third molars which had adjacent teeth. The complicated mandibular third molars^9^ refer to those impacted (partially or fully), or contacting the inferior alveolar canal, or occupying more than half of the overall bony thickness of the mandible, or having significant root hypertrophy or curve.

Based on the positions (Pell & Gregory’s classification), there were 25, 37 and 74 teeth in position A, B and C. Based on angulations (winter’s classification)^10^, there were 17 teeth vertical impacted, 37 mesial impacted, 59 horizontal impacted and 23 inverted impacted. All patients had undergone OPG and CBCT examinations.

### Risk Degree Classification

All teeth were categorized into 3 types according to the adjacent teeth resistances during the mesio-distal rotations of the impacted teeth after complete bone removal (bone removal to the apical level).

**Figure 1:**
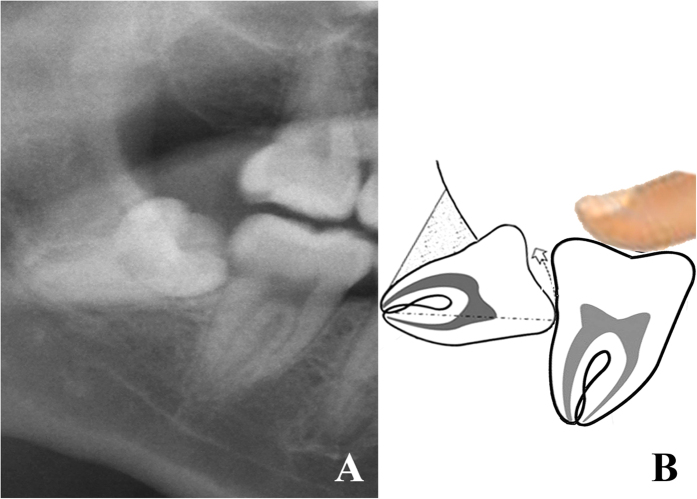
Teeth with risk degree I. (**A**) the OPG shows 48 was in risk degree I. (**B**) the sketch shows 48 had no adjacent tooth resistance after the complete bone removal.

Risk degree I: no adjacent tooth resistance (Fig. 1).

**Figure 2:**
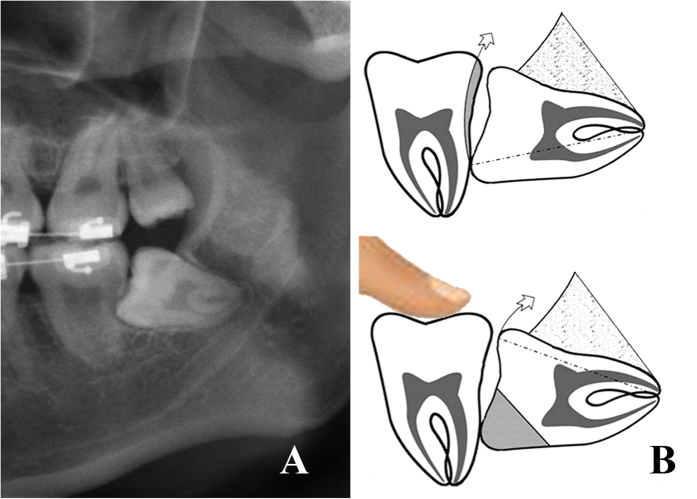
Teeth in risk degree II. (**A**) the OPG shows 38 was in risk degree II; (**B**) the sketch shows 38 got adjacent tooth resistance released after the complete bone removal and half crown sectioning.

Risk degree II: mild adjacent tooth resistance (no resistance after half crown sectioning) (Fig. 2).

**Figure 3:**
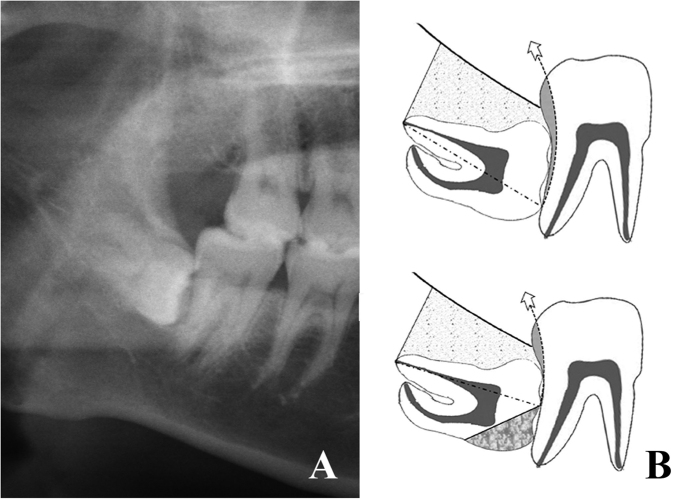
Teeth in risk degree III. (**A**) OPG shows 48 was in risk degree III. (**B**) the sketch shows 48 had adjacent tooth resistance after the complete bone removal and half crown sectioning.

Risk degree III: high resistance existed (still some resistance after half crown sectioning) (Fig. 3).

### Digital surgical simulations

Surgical simulation was performed in each case with the following steps: (1) the CBCT data were transferred into mimic software (Materialize Co, Leuven, Belgium) to reconstruct and differentiate the wisdom tooth, its adjacent tooth, the mandible, as well as the inferior alveolar canal; (2) the osteotomy lines were designed to remove adequate volume of bone; (3) the wisdom tooth was designed to rotate with or without tooth sectioning.

### Surgical procedures and overall outcomes evaluation

The surgery in each case was performed based on the preoperative digital simulation. The piezosurgery device(Piezotome2, Satelec, France) was applied in both bone removal and tooth sectioning.

Evaluation criteria include the success rate, the adjacent teeth mobility and pulp vitalities. Mobility assessment: degree I refers to the one with bucco-lingual mobility; degree II refers to the one with both bucco-lingual and mesio-distal mobilities; degree III refers to the one with bucco-lingual, mesio-distal and vertical mobilities. Pulp vitality testing (Elements Obsturation Unit, SybronEndo, USA) was only chosen for the adjacent teeth with vital pulps: the tested tooth was regarded abnormal when its measured value was different from the normal tooth (the difference should be > 10).

## Results

**Figure 4:**
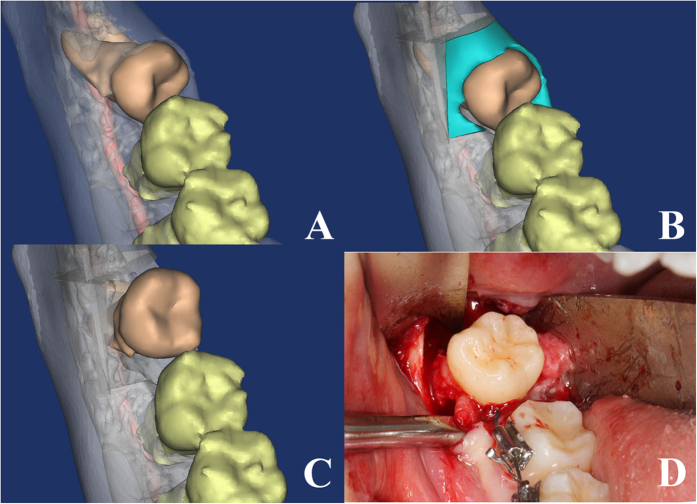
The extraction method of tooth in risk degree I. (**A–C**) three-dimensional reconstruction of CT helps the surgical simulation, which shows 48 could rotate mesio-distally after occlusal bone removal. (**D**) the critical surgical procedure.

**Figure 5:**
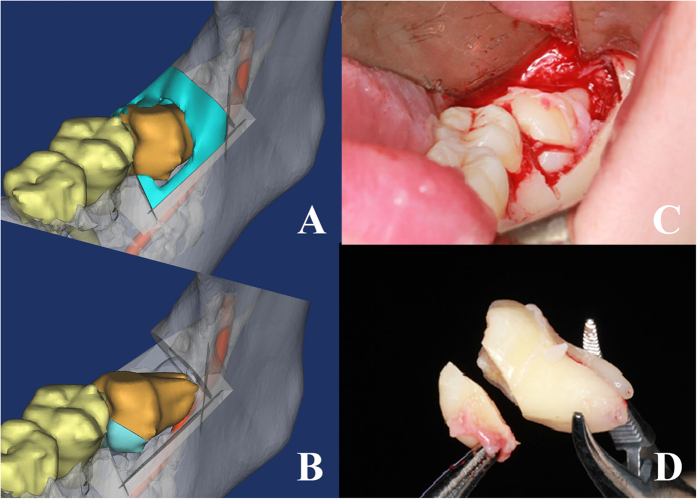
The extraction method of tooth in risk degree II. (**A,B**) surgical simulation shows 38 could rotate mesio-distally after the complete bone removal and mesio-half crown sectioning. (**C,D**) the critical surgical procedures.

**Figure 6:**
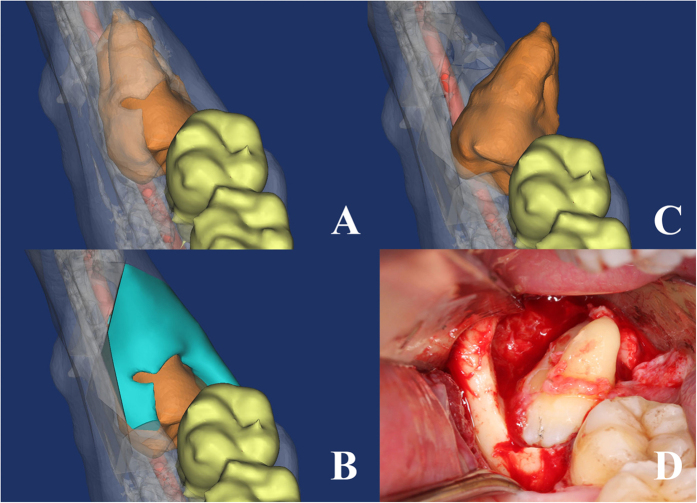
The extraction method of tooth in risk degree III. (**A–C**) surgical simulation shows 48 could rotate bucco-lingually. (**D**) the critical surgical procedure.

The general data of the patients are listed in Table 1. Among the 136 teeth, 37 teeth were in risk degree I and designed to rotate mesio-distally after complete bone removal (Fig. 4); 44 teeth were in risk degree II, with 38 of them designed to rotate mesio-distally with mesio-half crown sectioning (Fig. 5), and 6 designed to rotate bucco-lingually; 55 teeth were in risk degree III, with 36 designed to rotate bucco-lingually (Fig. 6), and 19 designed to rotate mesio-distally with crown-root sectioning.

The surgical procedure of each tooth was in accordance with the preoperative digital simulation. All wisdom teeth were extracted successfully with the exception of one adjacent tooth which was subluxated. All adjacent teeth were undamaged with normal morbility and/or normal pulp vitality.

## Discussion

According to our study, the complicated mandibular third molars were inclined to have higher adjacent teeth trauma risk (37 in degree I, 44 in degree II, 55 in degree III). This highlights the importance of paying more attention to this complication. The obstruction in the mesio-distal rotation path of the wisdom teeth is commonly accepted as adjacent teeth resistance, which is the most critical factor related to the risk degree. Based on this, we designed a risk degree classification to categorize complicated wisdom teeth. Using whether the wisdom teeth could rotate mesio-distally as a whole or with mesio-half crown sectioning as dividing basis, the classification has the surgical guidance value as well.

Piezosurgery is recommended in complicated impacted teeth extractions nowadays^4^,^5^. Compared with chisel and drill, the piezosurgry effects mainly on the mineralized tissues, thus the borders of the area affected can be determined accurately^11^. Besides, no char layer can be established through ultrasonic devices, leading to little thermal damage^12^. Due to its accuracy and safety, piezosurgery was used in our study for both osteotomy and tooth sectioning.

The methods to prevent adjacent teeth trauma include the removal of bone and tooth sectioning. With the exception of certain vertical impactions, teeth with incomplete bone removal will have the trend of forward moving^3^,^7^. This increases the adjacent teeth trauma risk. Besides, the bone resistance release could avoid complications like root fracture, which is worthy of attention in complicated wisdom teeth extraction. Tooth sectioning force increases the risk of inferior alveolar nerve damage and mandible fracture; thus it is suggested to use as a supplementary method in complicated wisdom tooth extraction. When the teeth are deeply impacted, changing the rotating direction is preferred to be chosen instead of tooth sectioning.

The extent of the bone removal, as well as the tooth sectioning could be designed preoperatively with the help of the digital surgical simulation. Digital surgical simulation was performed based on CBCT reconstruction with the advantages of adjustable transparency and flexible movements of different anatomic structures^13^. Digital template could be manufactured as well to guide the accurate osteotomy in the surgery^14^.

By reviewing the extractions, two methods were concluded in this study: the mesio-distal and buccal-lingual rotations. Tooth sectioning could assist either of them. Mesio-distal rotation includes crown and root rotation. The former refers to rotation in the sagittal plane using the apical region as the rotation center, while the latter refers to rotation in the sagittal plane using the contact area with adjacent tooth as the rotation center. Bucco-lingual rotation includes crown and root rotations in the horizontal plane with the same criteria in mesio-distal rotation.

Teeth in risk degree I are mainly those mesially/vertically impacted in positions A/B, which could be best extracted with mesio-distal rotations of the crowns. Teeth with risk degree II mainly consist of those mesially/horizontally impacted in positions A/B. Most teeth in risk degree II could be extracted with mesio-distal rotations with mesio-half crown sectioning (86.36%, 38/44). Some horizontally impacted teeth in degree II could be extracted with bucco-lingual rotations (13.64%, 6/44). Most of the teeth in risk degree III were horizontally/invertedly impacted in positions B/C, both having high adjacent teeth and bone resistances. Previous studies have reported three surgical methods to extract those teeth, including coronectomy^15^,^16^,^17^, tooth sectioning^7^, and lingual split technique^18^,^19^. The validity of the coronectomy is to be questioned due to the root left behind has the risk of infection. In the method of tooth sectioning, the sectioning forces to the deeply impacted teeth could lead to the IAN injury or mandible fracture. We recommend to rotate the wisdom tooth bucco-lingually with lingual split technique to release both bone and adjacent tooth resistance. In the bucco-lingual rotation methods, the root rotation is the best to avoid almost all the adjacent tooth resistance (Fig. 6). It is confirmed by our study, which shows teeth in degree III were usually extracted with bucco-lingual rotations (65.45%, 36/55), only a few horizontally impacted teeth in position C were extracted after sectioning (34.56%, 19/55).

In this study, the designed methods can contribute to the successful extraction of complicated wisdom teeth. One adjacent tooth was subluxated during the mesio-distal rotation of the impacted third molar (position A, mesially impacted) after the incomplete bone removal. Immediate fixation was done. Pulp vitality (1 year of follow-up) and tooth mobility (3 months of follow-up) were normal. The adjacent tooth trauma occurred because of the incomplete bone removal, which led to the forward movement of the wisdom teeth. The experience of this case shows the significance of complete bone removal.

This study has its limitation due to its retrospective character. In this study, the surgical design and digital simulation, as well as the surgeon’s skill contribute to the satisfied outcome. For other inexperienced surgeons, the extraction methods should be implemented with more precise design.

## Conclusion

The extraction methods dictated by the adjacent teeth resistances prevent adjacent teeth trauma. Digital simulation based on CBCT reconstruction has the surgical guidance value. Complete bone removal is of importance, and teeth with higher adjacent tooth trauma risks should consider bucco-lingual rotations.

## Additional Information

**How to cite this article**: Ye, Z.-X. *et al*. Adjacent tooth trauma in complicated mandibular third molar surgery: Risk degree classification and digital surgical simulation. *Sci. Rep.* **6**, 39126; doi: 10.1038/srep39126 (2016).

**Publisher's note:** Springer Nature remains neutral with regard to jurisdictional claims in published maps and institutional affiliations.

## Figures and Tables

**Table 1:** General data of the cases.

Study variable	Descriptive statistics
Sample size (case)	113 (136 teeth)
Sex: male (case)	50 (59 teeth)
location: left (case)	68
Age (years)	30.90 ± 11.53
Pell & Gregory classification (case)	
A	25
B	37
C	74
Winter’s classification (case)	
Vertical	17
Mesial	37
Horizontal	59
Inverted	23
